# Online Search Behavior for Cancer Immunotherapy Resources and Readability Analysis: An Opportunity to Aid in Medical Decision-making

**DOI:** 10.7759/cureus.5857

**Published:** 2019-10-07

**Authors:** Jie Deng, Ricky R Savjani, Percy Lee

**Affiliations:** 1 Department of Radiation Oncology, University of California Los Angeles, Los Angeles, USA

**Keywords:** cancer, immunotherapy, clinical trials, immune checkpoint, patient education, health literacy

## Abstract

Cancer patients are faced with increasing options for cancer care, especially with the introduction of cancer immunotherapy with immune checkpoint inhibitors (ICIs). Though many patients turn to online resources to supplement their decision-making, it is unknown whether online resources in cancer immunotherapy with ICIs are written at an appropriate level of readability according to national medical organizations. We performed a cross-sectional analysis of internet search behavior for cancer immunotherapy by ICIs and clinical trial availability per ClinicalTrials.gov in the United States (US) from 2004 - 2018 with subsequent quantitation of readability by four readability formulas of top 50 online resources. Internet search behavior for “cancer immunotherapy” has steadily increased since 2013 and coincides with the year of the US Food and Drug Administration (FDA) approval for individual ICIs. Furthermore, internet search behavior was significantly correlated with clinical trial availability in the US (R = 0.97, p < 0.0001). None of the top 50 resources available to patients were found to be within the recommended level of sixth-grade readability or less with only one (2%) written at the middle school level, 21 (42%) at the high school level, 23 (46%) at the university level, and five (8%) at a graduate level. Population-level internet search patterns may reflect patient behavior in seeking relevant online health information and may be influenced by new options for cancer therapy, including via clinical trials. However, low readability of available online resources may impede patient comprehension and negatively affect medical decision-making.

## Introduction

Clinical trials and subsequent United States (US) Food and Drug Administration (FDA) approval of immunotherapy with immune checkpoint inhibitors (ICIs) have created unprecedented treatment opportunities. Consequently, patients are faced with growing numbers of medical decisions.

The majority of patients turn to online resources, ranging from social media to federal websites, to gather health information to supplement decision-making [1]. Given that the average American adult reads between the sixth- and eighth-grade level [2], national organizations recommend that patient resources be written at the sixth-grade level or below [3-4]. However, analyses of patient-centered online health resources for cancer demonstrated that they may require a reading level of up to the 19th grade [5-7]. This gap in health literacy limits comprehension for decision-making, is associated with lower health outcomes, and is estimated to cost the US healthcare system up to $73 billion [4].

With the continued development of ICIs compounded with low clinical trial accrual, readability of related resources may become a particularly relevant issue. To our knowledge, this is the first study to specifically evaluate the readability of online resources for cancer immunotherapy with ICIs.

## Materials and methods

Internet search behavior

Internet search behavior on Google was evaluated for “cancer immunotherapy” and for each of the seven currently FDA approved ICIs using http://trends.google.com (Table 1). Behavior, quantified as search volume index (SVI), was extracted in January 2019. SVI is calculated by dividing each data point by total searches within set geography and time frame to illustrate relative popularity. All SVI data in the US were collected, spanning from January 1, 2004 to December 31, 2018.

**Table 1:** Food and Drug Administration (FDA)-approved Immune Checkpoint Inhibitors (ICIs) in the United States (US) Seven currently FDA-approved ICIs are listed by the molecular target, drug name, antibody clone names, and date of first FDA approval in the US. CTLA4: cytotoxic T-lymphocyte-associated protein 4; PD1: programmed cell death protein 1; PD-L1: programmed death-ligand 1.

Target	Drug name (clone names)	Trade name	Year of first FDA approval in the United States
CTLA4	Ipilimumab (BMS-734016, MDX010, MDX101)	Yervoy	2011
PD-1	Pembrolizumab, Lambrolizumab (MK-3475)	Keytruda	2014
	Nivolumab (BMS-936558, MDX-1106, ONO-4538)	Opdivo	2014
	Cemiplimab (REGN2810)	Libtayo	2018
PD-L1	Atezolizumab (MPDL3280A, MPDL328OA, RG7446)	Tecentriq	2016
	Avelumab (MSB0010718C)	Bavencio	2017
	Durvalumab (MEDI4736)	Imfinzi	2017

Available immunotherapy clinical trials with an immune checkpoint inhibitor

Available clinical trials with ICIs in “cancer” as the disease entity were extracted in January 2019 from ClinicalTrials.gov to evaluate whether the availability of treatment options with ICIs was correlated with online search patterns. The advanced search option was used for immunotherapy, immune checkpoint, and the seven FDA-approved ICIs by drug name (and former name), trade name, and antibody clones (Table 1). Geographical restriction to the US was the only restriction. Trial availability was determined by the “first posted” date starting from 2004 until 2018 to mirror the SVI analyses. Correlation of SVI and clinical trial availability was conducted with Pearson’s correlation.

Readability evaluation of online resources

A standard Google search of English websites was conducted for “cancer immunotherapy” on January 27, 2019. Resources directed towards researchers (including journal articles, conferences, workshops, research resources) and websites without original content (only hyperlinks) were excluded. Custom Python and Bash scripts (http://github.com/rsavjanimdphd/immunotherapyReadability) automated the extraction of website text and string tokenizer parsed sentences (Dridan R, Oepen S: Document parsing: towards realistic syntactic analysis. Presented at the 13th Internatl. Conf. on Parsing Technologies, Nara, Japan, Nov. 27-29, 2013). Each file was manually inspected, and readability measures were computed for each online resource. Total word count, words per sentence average, and four metrics of readability (Flesch-Kincaid Grade Level (FKGL) [8], Flesch Reading Ease Score (FRES) [9], Simple Measure of Gobbledygook (SMOG) [10], and the Gunning Fog Index (GFI) [11] were reported. The associated grade level required to understand the text was averaged.

## Results

Increasing internet search behavior for cancer immunotherapy correlates with increasing opportunities for treatment with ICIs

**Figure 1:**
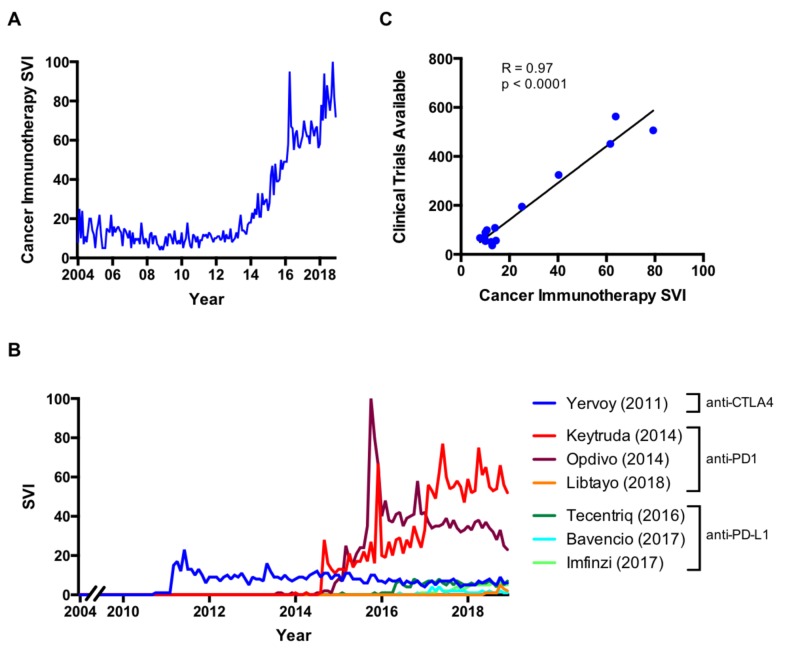
FDA approved immune checkpoint inhibitors (ICIs) in the United States A) SVI for “cancer immunotherapy” on Google; B) SVI for individual ICIs by trade name and drug class and year of first FDA approval; C) Correlation of “cancer immunotherapy” SVI with available clinical trials for ICIs on clinicaltrials.gov (R = 0.97, p < 0.0001). anti-CTLA4: anti-cytotoxic T-lymphocyte-associated protein 4; anti-PD1: anti-programmed cell death protein 1; anti-PD-L1: anti-programmed death-ligand 1; SVI: search volume index

The search volume index (SVI) for “cancer immunotherapy” has increased since 2013 (Figure 1A). Since cancer immunotherapy can include a wide variety of classes, we performed separate analyses for individual FDA-approved ICIs (Table 1). Analyses were carried out by trade name since trade names are more frequently used than generic names [12], though trade and generic names showed similar trends (data not shown). These additional analyses demonstrated that the search pattern was temporally associated with FDA approval for each drug (Figure 1B). Furthermore, we found that “cancer immunotherapy” SVI was strongly and positively correlated with clinical trials available for ICIs (R = 0.97, p < 0.0001) (Figure 1C).

Online health resources require a reading level above recommended levels

**Figure 2:**
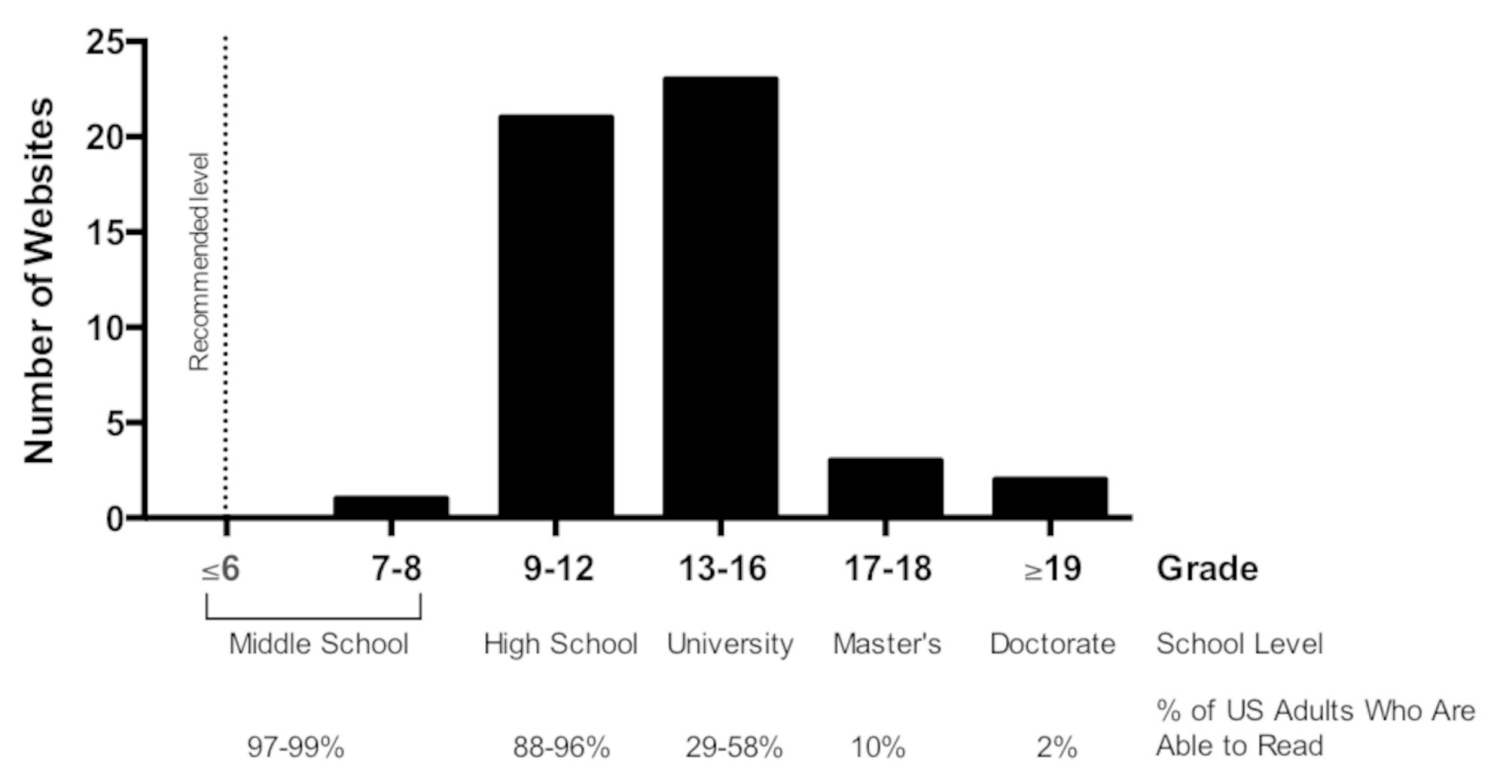
Distribution of required reading level for the top cancer immunotherapy websites on Google The grade level for each website was determined the average of FRES, FKGL, GFI, and SMOG formulas. School-level and percentage of United States (US) adults who can read at each respective grade level is displayed. The dashed line represents the recommended reading level for patient resources by national organizations. FKGL: Flesch–Kincaid Grade Level; FRES: Flesch Reading Ease Score; GFI: Gunning Fog Index; SMOG: Simple Measure of Gobbledygook

The top 50 websites for “cancer immunotherapy” relating to ICIs returned from a standard Google (http://www.google.com) inquiry were analyzed by four separate reading scales, including SMOG, the formula recommended by the National Cancer Institute (NCI) (Table 2) [13]. The mean of all four readability assessments was calculated to mitigate variability between formulas for all 50 websites (Figure 2). None of the 50 websites were written at the recommended sixth-grade level or below with one (2%) written at the middle school level, 21 (42%) at the high school level, 23 (46%) at the university level, and five (8%) at a graduate level.

**Table 2:** Results of Readability Analysis of Top 50 Cancer Immunotherapy Websites Word count, words per sentence and readability scores for four distinct formulas. FKGL: Flesch–Kincaid Grade Level; FRES: Flesch Reading Ease Score; GFI: Gunning Fog Index; SD: standard deviation; SMOG: Simple Measure of Gobbledygook

	Mean	SD	Range
Word count	956.3	982.18	116 – 5046
Words per sentence	21.43	4.86	14.34 – 40
FRES	51.72	14.69	9.15 – 79.90
FKGL	11.37	2.91	5.72 – 20.37
GFI	14.53	2.79	7.91 – 21.00
SMOG	12.66	1.84	7.78 – 16.21

## Discussion

The shift from a paternalistic model of medicine to one that is patient-centered has allowed patients to become more involved in their treatment [14]. This model of shared decision-making, coupled with increasing therapeutic options, constantly creates a new landscape that patients must navigate with their physician.

Immunotherapy by ICIs is a clear example of how this landscape has changed for cancer patients. Steadily increasing SVI for cancer immunotherapy further supports the broad interest in these therapeutics. SVI was significantly and strongly positively correlated with clinical trial availability with ICIs, suggesting that the availability of treatment may influence patient health information-seeking behavior on the internet.

Despite recommendations for readability at the sixth-grade level or below for patient resources [3], we found that none of the top 50 websites associated with ICIs met that criteria, with 98% requiring high school level education or above. However, even at a high school level, only a minority are proficient in science literacy [15]. Collectively, this highlights an additional obstacle patients face when participating in shared decision-making with their physician. Poor understanding of ICIs may also function as an additional barrier to clinical trial enrollment. This represents an important opportunity for physicians to review patient understanding of their independent research.

Our findings are consistent with the evaluation of other online cancer resources [5-7], although this is the first to specifically address cancer immunotherapy among English websites. Study limitations include evaluation of search behavior restricted to Google, which may not be generalizable, although this represents the most popular search engine in the US [16]. Other limitations include inherent biases within the formulas that preferentially assign difficulty to syllables and sentence length and do not account for other complexities, such as syntax and diction, or consider other factors, such as website design, that may affect comprehension. Nevertheless, these formulas allow quantitation of readability and mirroring previously published sets of readability formulas enable comparison to preexisting literature.

These data also suggest that increasing the availability of clinical trials and treatment options may influence online search behavior for related therapies. With both a possible increase in clinical trials [17] and patient eligibility [18], the ability for patients to understand health information online may become increasingly more important.

## Conclusions

We recommend possible avenues of improvement to include the revision of existing materials to meet readability recommendations and supplementation of written information with additional visual and auditory components to engage more dimensions of learning and comprehension. As patients continue to use the internet to find resources regarding ICIs, improved readability may help patients better understand treatment options available and may also facilitate clinical trials accrual.
